# A simple and robust method for laboratory-scale preparation of butter

**DOI:** 10.3168/jdsc.2024-0571

**Published:** 2024-06-28

**Authors:** Sandra Beyer Gregersen, Lise Margrethe Boesgaard, Dionysios D. Neofytos, Mads Eg Andersen, Ulf Andersen, Milena Corredig

**Affiliations:** 1 Department of Food Science, Aarhus University, 8200 Aarhus, Denmark; 2 Arla Innovation Centre, Arla Foods, 8200 Aarhus, Denmark

## Abstract

The aim of this study was to develop a small-scale model system resembling the micro- and meso-structure of butter, namely having a water droplet size distribution and water content close to that of commercially produced butter. Although it is possible to churn cream on a small scale, matching the microstructure of butter is a challenge. A 2-step churning process was introduced with the application of a kitchen mixer. The resulting microstructure was evaluated using confocal laser scanning microscopy. In addition, low-field nuclear magnetic resonance was used to determine the water droplet size distribution. Results demonstrated that a water content of 16% to 19% could be obtained with the proposed procedure, close to the standard water content of 16%. Average water droplet size ranged between 2.5 to 4.3 µm and did not depend on batch-to-batch variations, nor storage-induced differences in the cream. In conclusion, the proposed method could be employed to prepare water-in-oil emulsions with a microstructure similar to that of butter and opens new opportunities for studying microbial growth, flavor release, and texture formation.

## Highlights

• The small-scale model system resembled the micro- and meso-structure of butter.
• This study used a 2-step churning process using a kitchen mixer.
• Draining after phase inversion prevents re-incorporation of buttermilk.
• The water content and water droplet size were comparable to that of commercial butter.
• Batch-to-batch variations and storage-induced differences had no effect on the cream.

## Graphical Abstract

**Figure:**
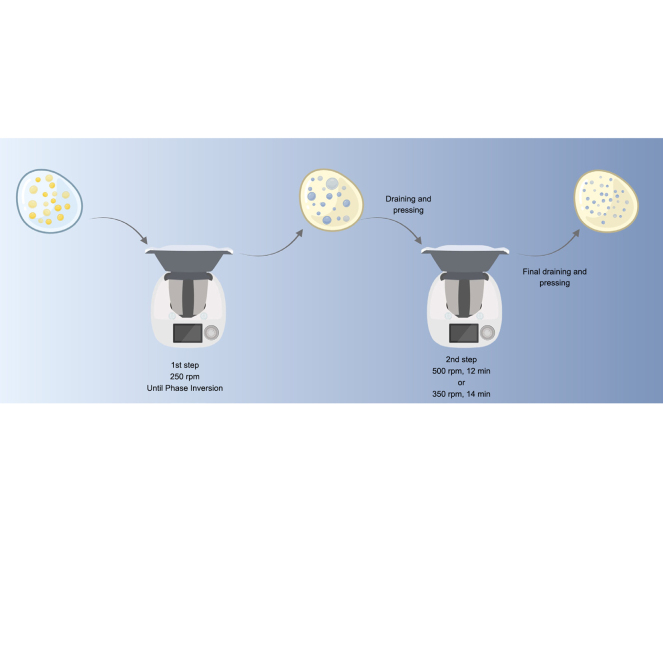
**Summary:** The objective of this study was to develop a small-scale model system resembling the micro- and meso-structure of butter. It proposes a 2-step process for cream churning using a kitchen mixer. The first step involved churning at 250 rpm until phase inversion, followed by removal of buttermilk. The second step included churning at 500 rpm for 12 minutes or 350 rpm for 14 minutes before final draining and pressing. The resulting microstructure was evaluated using confocal laser scanning microscopy. Low-field nuclear magnetic resonance was used to determine the water-droplet size distribution, and hardness was evaluated by texture analysis. The resulting water content and microstructure resembled that of a commercial butter sample and did not depend on batch-to-batch variations or storage-induced differences in the cream. Figure created with BioRender.com.

Butter is obtained by phase inversion of cream, an oil-in-water emulsion, which by churning is transformed into a water-in-oil emulsion. The resulting emulsion contains at least 80% (wt/wt) milk fat which is partially crystallized, and no more than 16% (wt/wt) water, homogeneously dispersed in small droplets. The size distribution of the water phase affects the chemical and physical stability of the final product, as well as its organoleptic properties, and therefore, it is an important parameter for the control of microbiological growth (Rønholt et al., 2013; Panchal and Bhandari, 2020). For instance, the droplet size should be kept as low as possible since larger droplets can serve as growth zones for bacteria (Michelon et al., 2016). However, good laboratory-scale model systems matching the microstructure of industrially produced butter, for example, with varying droplet size or composition, do not exist, and therefore predictive models cannot be developed. In other words, expanding the knowledge of butter quality can only rely on full-scale or at least pilot plant–based formulations. These approaches are time-intensive, demand large amounts of material, and most important are often restricted by constraints on testing parameters, even when at the pilot scale. In addition to being more flexible, for example, allowing to test emerging pathogenic strains or non-food-grade ingredients, the development of laboratory models is also important to reduce food waste. A reliable laboratory model can also open new possibilities for studying flavor release and texture formation in butter systems.

Simple small laboratory-scale churns have been used in the past, but mainly to induce phase inversion and produce buttermilk (Corredig and Dalgleish, 1998), and the properties of the resulting butter are not reported. Food mixers can serve as a simple unit for phase inversion and butter production on a small scale and have been used to study process-induced changes in the fat crystal network of butter (Rønholt et al., 2012; Lee and Martini, 2018). However, these studies have not aimed at optimizing and controlling water content or water droplet size distribution, which often either show high variation or are not reported.

The aim of this study was to develop a robust method for laboratory-scale production of butter to obtain a microstructure comparable to that of industrially produced butter with focus on the water content and droplet size. It was hypothesized that by implementing a 2-step churning process to prevent the re-incorporation of buttermilk into the structure after phase inversion, it is possible to further reduce water content and improve control of the droplet size.

A Thermomixer (Vorwerk & Co KG, Germany) was used for churning of cream (500 mL, precooled to 9.0°C) by first mixing the cream (250 rpm) until phase inversion had taken place, as determined visually. The serum phase (buttermilk) was then removed by draining with a cheesecloth. A second mixing was then performed using high speed for a short time (500 rpm for 12 min) or a low speed for a longer time (350 rpm for 14 min). These 2 conditions were selected based on preliminary screening results (data not shown), which also demonstrated the necessity for a compromise between speed and treatment time. The temperature during processing was monitored and did not exceed 20°C in any case. After the second churning step, the buttermilk was again separated from the butter mass by the application of a sieve. The butter mass was then stored for 1 h in the refrigerator (4°C) before undergoing a final pressing using a manual press covered with a cheesecloth. To probe the robustness of the method, 2 different creams (38.7% fat and 39.7% fat) were used as starting material after storage at 4°C for either 2 or 7 d. A commercial butter sample was used for comparison (Lurpak unsalted butter, Arla Foods amba, Denmark) and analyzed in a similar manner as the small-scale-produced samples. The water content of all samples was determined using a moisture analyzer at 110°C (HR73 Halogen Moisture Analyzer, Mettler Toledo, Greifensee, Switzerland). The butter microstructure was evaluated by confocal laser scanning microscopy (**CLSM**) employing a Nikon Eclipse T*i*2 microscope with a Nikon Plan Fluor 40x/1.30 Oil objective. Fluorescein isothiocyanate (**FITC**) and Nile Red were used for visualization of proteins in the water phase and fat, respectively, as described by Buldo and Wiking (2012). In short, dyes dissolved in acetone were applied to the microscopy slide, allowing time for the acetone to evaporate before samples were left in contact with the dyes for at least 10 min before imaging.

Hardness was determined by textural analysis (Stable Micro Systems TA-XTplus100C) with a conical probe (SMS P/60C, Bruker) at the force at 8 mm (speed of 0.2 mm/s). The water size distribution was analyzed using low-field nuclear magnetic resonance (**NMR**; Minispec mq20 Analyzers, Bruker, Germany) equipped with a pulsed gradient field unit (20 MHz equipped with a 4630_10AVGX probe) and operating at 5°C, by using the self-diffusion coefficient of 1.31 × 10^−9^ m^2^/s in water, reporting the mean droplet size and percentage of droplets >10 µm. The data analysis assumes the presence of spherical water droplets and a log-normal distribution of their size.

All experiments were performed in duplicate, and data are presented as averages with SD. Statistical significance at a *P* < 0.05 level was determined using ANOVA (factors: treatment, cream fat content, and age of cream).

Table 1 summarizes the impact of the 2 shear conditions tested during churning on the water content and size distribution for the samples produced using cream stored at 4°C for 2 or 7 d before churning. The water content of all small-scale-produced samples ranged between 16% and 19% water, with the fat content being assumed to be inversely related. This was slightly higher than the water content of the butter of commercial origin. The average water droplet size for the commercial sample was 2.1 ± 0.04, with no detectable population larger than 10 µm. The small-scale-produced samples also showed a small average water droplet size (2.7 to 4.2 µm) but with varying percentages of larger droplets. This might be ascribed to the nature of the NMR measurements, which can be influenced by the presence of large water pockets. These can arise either from an initial heterogeneous structure or from the fusion of water droplets during preparation.

**Table 1:** Properties of laboratory-scale butter in terms of water content and water droplet size measured with low-field NMR, and hardness from texture analysis^1^

Second churning	Cream		Water content (%)	Water droplet size		Hardness (N)
	Age (d)	Fat (%)		Mean (μm)	>10 μm (%)	
High speed, short time	2	39.7	17.4 ± 1.2^a^	3.2 ± 0.3^a^	6.7 ± 5.1^a^	10.5 ± 0.6^ab^
High speed, short time	2	38.7	16.1 ± 1.1^a^	2.7 ± 0.3^a^	2.0 ± 1.1^a^	17.3 ± 1.0^ab^
High speed, short time	7	39.7	17.0 ± 2.0^a^	3.6 ± 0.3^a^	8.8 ± 3.0^a^	17.6 ± 3.1^ab^
High speed, short time	7	38.7	16.1 ± 0.2^a^	2.5 ± 0.1^a^	1.5 ± 0.2^a^	7.9 ± 0.3^ab^
Low speed, long time	2	39.7	19.2 ± 0.8^a^	4.2 ± 0.8^a^	17.1 ± 5.0^a^	9.1 ± 0.8^ab^
Low speed, long time	2	38.7	17.3 ± 0.1^a^	4.0 ± 0.3^a^	14.9 ± 5.8^a^	13.0 ± 0.8^b^
Low speed, long time	7	39.7	17.2 ± 0.1^a^	3.4 ± 0.1^a^	9.4 ± 2.6^a^	11.9 ± 1.7^ab^
Low speed, long time	7	38.7	16.1 ± 0.7^a^	3.3 ± 0.3^a^	9.5 ± 2.7^a^	5.8 ± 1.8^a^
Commercial butter sample			15.7 ± 0.1	2.1 ± 0.04	0	21.2 ± 3.2

a,b Different lowercase letters indicate significant (*P* < 0.05) differences within each column.

1 Samples were obtained using 2 different creams (38.7% or 39.7% fat), aged for 2 or 7 d, and churned using a 2-step process. The first step involved churning at 250 rpm until phase inversion, followed by removal of buttermilk. The second step included churning at 500 rpm for 12 min (high speed and short time) or 350 rpm for 14 min (low speed and long time) before final draining and pressing. Analysis of commercial butter included for comparison. Data represent averages with corresponding SD.

Table 1 also shows the hardness of the resulting butter highlighting large variation and a softer texture for all treatments compared with the control. The focus of this work was to develop a model system with low water content and small water droplet size averages, and therefore the texture was not further optimized. However, by controlling the development of the fat crystal network, which is a key factor determining the texture of butter, it may be possible to also reach comparable texture conditions. Here, processing conditions to consider would be the temperature history of the cream, the temperature during processing, agitation, and subsequent storage (Rønholt et al., 2012; Lee and Martini, 2018).

Figure 1 shows representative CLSM images of the microstructure obtained. All laboratory-scale produced samples showed a homogeneous distribution of small water droplets, regardless of the treatment applied. These observations confirmed the low-field NMR analysis summarized in Table 1. In general, the microstructure resembles that observed in the commercial butter sample, but with the presences of slightly larger water droplets in some cases. It can however be noted that no large water domains were observed in the CLSM images as shown by the NMR measurements. This discrepancy might be due to that on the one hand the microscopy only represents a local area, whereas NMR provides a bulk measurement, and on the other hand the NMR is subjected to large water pockets as described above, which is not the case with CLSM. Furthermore, CLSM images for samples obtained by application of high speed for a short time suggested the presence of slightly larger droplets for creams with a high-fat content (Figure 1B and D); however, this was not shown by low-field NMR. To ensure the robustness again batch-to-batch variation, a longer mixing time at a lower speed may therefore be preferred during laboratory churning.

**Figure 1:**
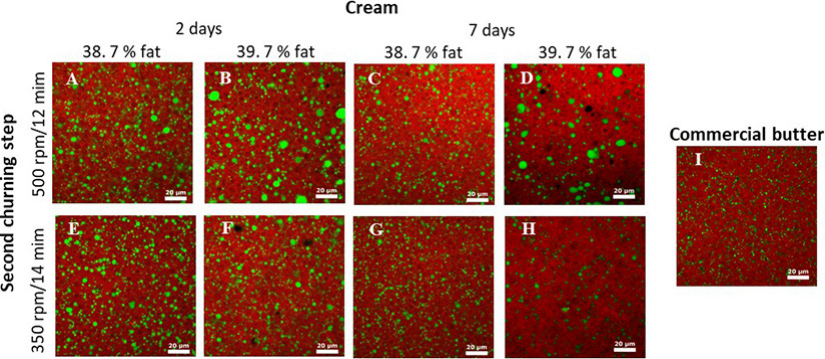
Representative images of the microstructure of laboratory butter samples obtained by churning at 500 rpm for 12 min (A–D) or 350 rpm for 14 min (E–H), for 2 different creams with either 38.7% fat (A, C, E, G) or 39.7% fat (B, D, F, H) stored for either 2 or 7 d before processing. All experiments were performed with a 2-step churning process including removal of water after phase inversion followed by storage for 6 d before analysis by confocal laser scanning microscopy identifying protein representing water droplets (green) and fat (red) droplets by specific fluorescence dyes. Representative image of a commercial butter (Lurpak) is shown for comparison (I).

In conclusion, this experimental approach established a simple and reproducible model system for laboratory-scale butter preparation, which can provide a platform for evaluating microbial growth, flavor release, or texture formation.
